# Diagnosis and treatment of influenza based on health insurance claims between the 2010–2011 and 2019–2020 influenza seasons in Japan

**DOI:** 10.1111/irv.12977

**Published:** 2022-03-17

**Authors:** Eiko Shimizu, Kosuke Iwasaki, Yoshie Hongo, Manami Yoshida, Masahiro Kinoshita, Shinzo Hiroi, Daisuke Tamura

**Affiliations:** ^1^ Social Cooperation Program of IT Healthcare, Graduate School of Pharmaceutical Sciences The University of Tokyo Tokyo Japan; ^2^ Milliman Inc. Tokyo Japan; ^3^ Medical Affairs Department, Integrated Disease Care Division Shionogi & Co., Ltd. Tokyo Japan; ^4^ Department of Pediatrics Jichi Medical University Tochigi Japan

**Keywords:** antiviral agents, COVID‐19, diagnosis, diagnostic tests, healthcare administrative claims, influenza

## Abstract

Medical practices for influenza virus infection vary among countries. In Japan, treatment with anti‐influenza drugs is recommended for patients diagnosed with influenza. This health claims database study provides quantitative information aimed at describing the actual medical practices, including diagnostic testing and medication use, for managing influenza in Japan. Most patients diagnosed with influenza underwent diagnostic tests and were prescribed anti‐influenza drugs. Meanwhile, the majority of patients prescribed anti‐influenza drugs had undergone diagnostic testing. However, an increase in the percentage of anti‐influenza prescriptions without diagnostic testing was observed during the 2019–2020 influenza season, which may be associated with the COVID‐19 pandemic.

## 1 INTRODUCTION

Although influenza virus infection is self‐limiting in most patients, it is an important disease that affects many people and causes severe symptoms in some cases. In Japan, the epidemic occurs mainly during winter, and the cumulative seasonal number of influenza patients is approximately 5%–15% of the population.^1^ Influenza can cause complications such as influenza‐associated encephalopathy in infants and young children and pneumonia in the elderly.^2^, ^3^

Medical practices for influenza vary among countries, although rapid diagnosis and clinical management reduce the incidence of severe illness and complications.^4^ In Japan, treatment with anti‐influenza drugs is recommended for patients who were confirmed or suspected of having influenza by the Japanese Association for Infectious Diseases (JAID).^5^ A rapid diagnostic test is usually performed for suspected cases, but the sensitivity varies depending on time between symptom onset and presentation.^6^ Consequently, influenza diagnosis may not always be based solely on test results but can instead be comprehensively determined based on symptoms and disease prevalence.

Quantitative information about medical practices for influenza, including diagnostic testing, diagnosis, and treatment, is not well provided in Japan, although the epidemic status of seasonal influenza has been reported by surveillances,^2^, ^7^, ^8^ and a high prescription rate (98.4%) for hospitalized child patients during the 2009 influenza A (H1N1) pandemic has been reported by a chart review study.^9^ Furthermore, medical practices may have changed due to the coronavirus disease 2019 (COVID‐19) pandemic. Therefore, this study analyzed insurance claims databases to show the quantitative information about medical practices for influenza between the 2010–2011 and 2019–2020 seasons and better understand the medical handling of influenza in Japan.

## 2 METHODS

A claims database of Japanese health insurance associations,^10^ provided by JMDC Inc., contains data of employees and their family members obtained from companies belonging to these associations, which corresponded to approximately 6% of the Japanese population as of August 2020. The percentage of males is higher than that of females, people aged ≥65 years are few (those aged 70–74 are <1% of the members), and those aged ≥75 years are excluded. The database includes the daily records of medical practices, including diagnostic tests, treatments, and monthly diagnostic records. All records associated with remuneration can be traced for each individual, regardless of medical institution type. Results of diagnostic tests are not included.

This study used descriptive statistics. We analyzed patient numbers and the distribution of combination patterns of diagnostic tests (hereafter tests), diagnoses, and prescriptions for anti‐influenza medications throughout each season. We also analyzed weekly numbers of tests and prescriptions. The study period was from September 2010 to April 2020 and was divided by each influenza season, starting from September 1 of a current year and ending on August 31 of the following year (total, 52 weeks). The 2019–2020 season (2019/2020) lasted until April 2020. Tests were defined as a “qualitative influenza virus antigen test”; the diagnoses included definitive diagnoses coded as J09, J10, or J11 by the International Statistical Classification of Diseases and Related Health Problems (10th revision)^11^; prescriptions included the following anti‐influenza drugs: oseltamivir phosphate, zanamivir hydrate, baloxavir marboxil, peramivir hydrate, or laninamivir octanoate hydrate. We defined all anti‐influenza drug prescriptions as those indicated for the treatment of influenza because prophylactic prescriptions were not covered by Japanese health insurance.

We used the SAS software package, version 9.4 (SAS Institute, Cary, NC, USA) and Microsoft Excel 365 (Microsoft, Redmond, WA, USA) for data analysis.

## 3 RESULTS

**FIGURE 1:**
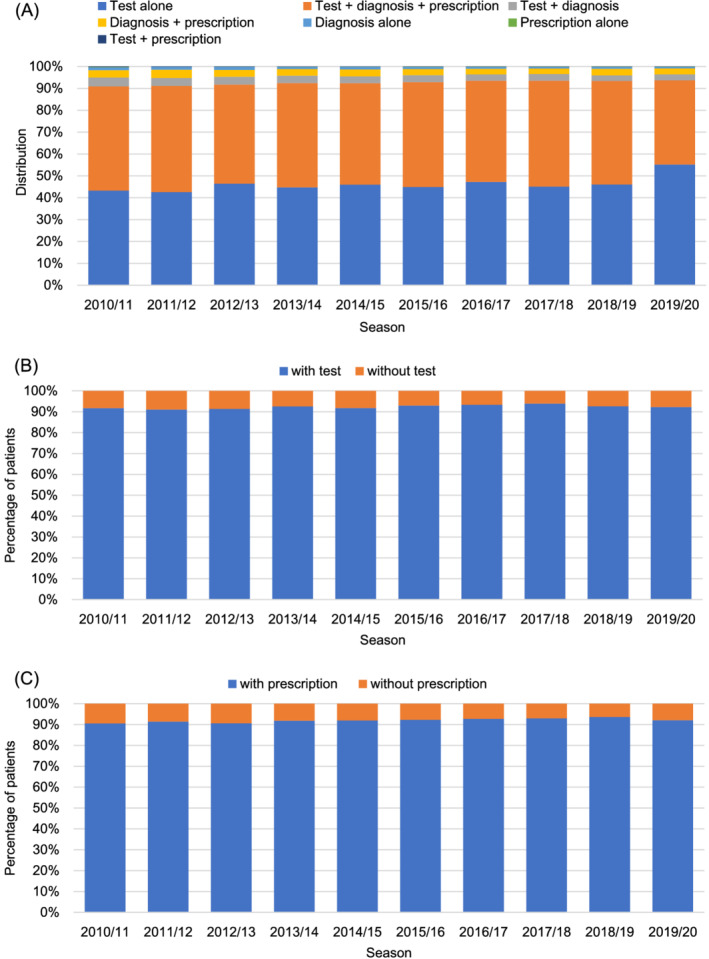
Combination patterns of medical operations for influenza by season (2010/2011–2019/2020 season). (A) Distribution of test, diagnosis, and prescription for influenza. (B) Percentage of patients who took tests out of those diagnosed with influenza. (C) Percentage of patients prescribed anti‐influenza drugs for influenza out of those diagnosed with influenza

The percentage of patients with records of tests, diagnoses, or prescriptions for influenza was 11.0% of the population in 2012/2013—the lowest during the study period (Table 1). In 2017/2018 and 2018/2019, the percentage was the highest, almost 20% in both the seasons. Among the combination patterns, the test + diagnosis + prescription combination was the most frequent, followed by test alone until 2018/2019, but test alone was the most frequent (55.2%) in 2019/2020 (Figure 1A). Among patients with a diagnosis, ≥90% took the test (Figure 1B) and ≥90% received prescriptions (Figure 1C) during all seasons.

**TABLE 1:** The number and demographics (average and standard deviation) of individuals in the population in the database and percentage of patients who received a test, diagnosis, or prescription for influenza infection in each influenza season

Season	2010/2011	2011/2012	2012/2013	2013/2014	2014/2015	2015/2016	2016/2017	2017/2018	2018/2019	2019/2020
Number of individuals in the population	1,317,567	1,765,080	2,771,476	2,956,608	4,133,660	5,149,566	6,116,093	7,179,776	7,457,659	6,722,866
Age (average)	31.4	32.0	32.1	32.3	33.1	33.4	33.5	33.5	33.7	34.1
Age (SD)	17.9	18.2	18.2	18.3	18.5	18.6	18.5	18.4	18.4	18.6
Female (%)	44%	44%	44%	44%	45%	45%	46%	46%	46%	45%
Patients with test, diagnosis or prescription (%)	13.5%	14.9%	11.0%	15.8%	12.9%	15.4%	16.9%	19.7%	19.5%	14.8%

Abbreviation: SD, standard deviation.

**FIGURE 2:**
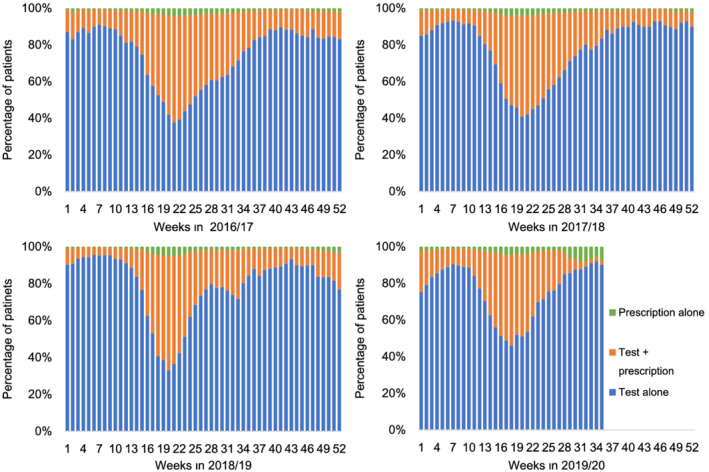
Combination patterns of test and prescription for influenza on weekly basis by season (2016/2017–2019/2020 season). Each season lasted from September 1 of a current year until August 31 of the following year

In the last four seasons, the number of patients who had either a test or prescription was the highest during the 21st or 22nd week for three consecutive seasons from 2016/2017 and in the 17th week in 2019/2020 (Figure S1). Among the combination patterns, the test + prescription combination was approximately 50%–60% around the week with the highest number of patients, and test alone was the highest in the other weeks (Figures 2 and S1). Prescription alone was <5% in most weeks. In particular, the percentage of prescription alone was low during the weeks when the number of patients was small with a high percentage of test alone, and the percentage was high when the number of patients was large with a high percentage of the test + prescription combination. However, in 2019/2020, the percentage of prescription alone was high and remained approximately 6%–8% from the 29th to the 35th week, although the patient number was small (Figures 2 and S1).

## 4 DISCUSSION

We examined the combination patterns of medical practice for influenza by season between 2010/2011 and 2019/2020. Among all combinations of three categories (tests, diagnoses, and prescriptions), the combination of test + diagnosis + prescription was the most common except 2019/2020. This combination was 40%–50% until 2018/2019, although the percentage of the patients with any of them among population varied (11%–20%).

However, in 2019/2020 (until April), the category of test alone was approximately 10% higher than that in the other seasons. Regardless of these differences, most patients diagnosed with influenza underwent diagnostic tests and received anti‐influenza drugs every season. The JAID emphasizes the importance of starting treatment early in the course of illness and recommends treatment initiation in cases of confirmed or suspected influenza.^5^ Our results suggest that the practice is consistent with this policy for patients diagnosed with influenza at the study sites. Nevertheless, these results excluded patients without a diagnosis, including those with influenza‐like symptoms, which were not accurately recorded in the database. Even so, considering a Japanese online survey reporting that 75.5% of people visited medical institution when having influenza‐like illness,^12^ our study might include the majority of those with symptoms in Japan. Most patients who were prescribed drugs also underwent testing; however, patients who were prescribed anti‐influenza drugs for influenza without a test were more frequently observed from the 29th week in 2019/2020. This might be explained by the influence of messaging requesting prescription based on clinical diagnosis alone from the Japanese Medical Association in March 2020 as a countermeasure for the COVID‐19 pandemic.^13^ Later, in August 2020, proposals from the same association released the recommendation of diagnostic tests for both influenza and COVID‐19.^14^ Therefore, this observation may have changed again in late 2020.

This study had several limitations. First, diagnosis codes may have been intentionally assigned along with diagnostic tests or prescription drugs. In addition, patients who visit medical institutions are usually willing to take treatment. These might contribute to high percentages of the combinations of diagnoses + tests and/or prescriptions. Second, the database included few elderly people, which did not accurately reflect the Japanese population ratio. Third, we defined medical practice based on claims records; consequently, the accuracy of the results relies on the accuracy of the records. Ultimately, the claims for test and treatment are assumed to be accurately recorded because they are generated to claim medical care fees. However, prescriptions uncovered by the medical insurance system, such as prophylactics, could be included in the record if diagnosis was made to prescribe it. Fourth, the database had no information on diagnostic test results; therefore, we could not examine whether the diagnosis was based on the test results. During the epidemic, even a patient with a negative result might be diagnosed with influenza based on comprehensive judgment and then given treatment. Lastly, this study analyzed patients who visited medical institutions and did not include those who had influenza‐like symptoms but not visited.

## 5 CONCLUSION

This study indicates that most patients diagnosed with influenza underwent diagnostic tests and were treated with anti‐influenza drugs. Further studies are needed to evaluate whether the patients were tested and treated for influenza from early onset. Since the COVID‐19 pandemic began, the status of diagnosis and treatment of influenza may have changed according to social situations and recommendations from academic associations. Continuous monitoring of the actual status of treatment and diagnosis may be necessary to provide information to deal with influenza.

## AUTHOR CONTRIBUTIONS

**Eiko Shimizu:** Conceptualization; methodology; project administration. **Kosuke Iwasaki:** Conceptualization; formal analysis; methodology; software. **Yoshie Hongo:** Conceptualization; formal analysis; methodology; project administration. **Manami Yoshida:** Conceptualization; methodology. **Masahiro Kinoshita:** Methodology. **Shinzo Hiroi:** Conceptualization; methodology. **Daisuke Tamura:** Conceptualization; methodology.

## PATIENT CONSENT STATEMENT

Informed consent was not required because this study used a database that contained anonymized data collected for secondary use.

## PERMISSION TO REPRODUCE MATERIAL FROM OTHER SOURCES

This manuscript does not include any previously published material.

### PEER REVIEW

The peer review history for this article is available at https://publons.com/publon/10.1111/irv.12977.

## Supporting information




**FIGURE S1** Number of patients receiving either test or prescription for influenza on a weekly basis by season (2016/2017–2019/2020 season). Each season lasted from September 1 of a current year until August 31 of the following year.

## Data Availability

The data that support the findings of this study are available from JMDC Inc. Restrictions apply on the availability of these data, which were used under license for this study. Data are available from the authors with the permission of JMDC Inc.
