# Supplementary material for: Diagnosis and treatment of influenza based on health insurance claims between the 2010–2011 and 2019–2020 influenza seasons in Japan
Source: Influenza Other Respir Viruses. 2022 Mar 17;16(4):621–5. doi: 10.1111/irv.12977 (PMC9178068; doi:10.1111/irv.12977)
Supplement: Supplementary file 1 — FIGURE S1 Number of patients receiving either test or prescription for influenza on a weekly basis by season (2016/2017–2019/2020 season). Each season lasted from September 1 of a current year until August 31 of the following year. [file IRV-16-621-s001.pptx]

## Slide 1
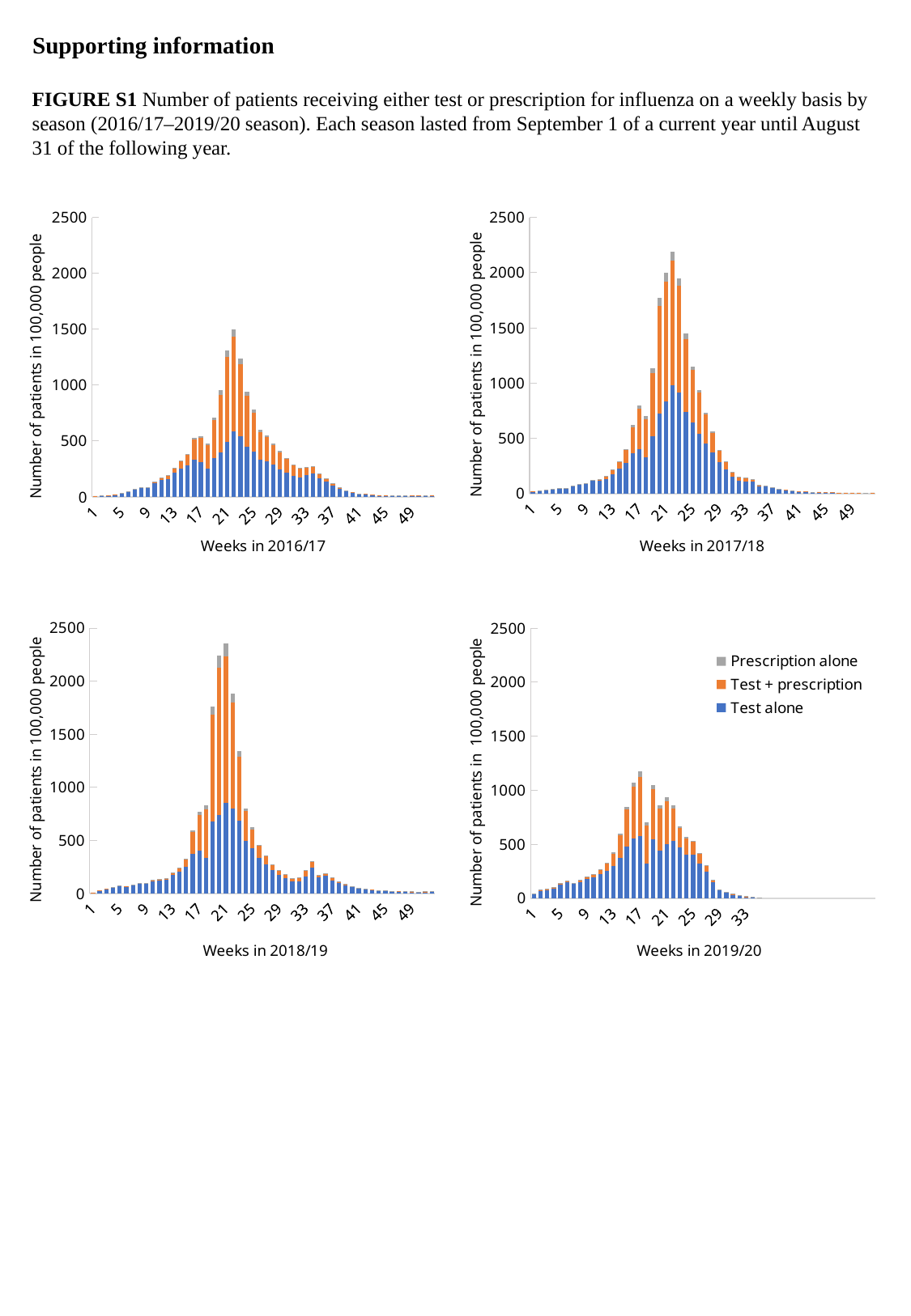

Supporting information
FIGURE S1 Number of patients receiving either test or prescription for influenza on a weekly basis by season (2016/17–2019/20 season). Each season lasted from September 1 of a current year until August 31 of the following year.
### Chart
| Category | 検査のみ | 検査かつ処方 | 処方のみ |
|---|---|---|---|
### Chart
| Category | 検査のみ | 検査かつ処方 | 処方のみ |
|---|---|---|---|
### Chart
| Category | 検査のみ | 検査かつ処方 | 処方のみ |
|---|---|---|---|
### Chart
| Category | Test alone | Test + prescription | Prescription alone |
|---|---|---|---|
